# Supplementary material for: Bioorthogonal labeling of transmembrane proteins with non-canonical amino acids unveils masked epitopes in live neurons
Source: Nat Commun. 2021 Nov 18;12:6715. doi: 10.1038/s41467-021-27025-w (PMC8602626; doi:10.1038/s41467-021-27025-w)
Supplement: Supplementary file 1 — Supplementary Information [file 41467_2021_27025_MOESM1_ESM.pdf]

Supplementary Information for

Bioorthogonal labeling of transmembrane proteins with non-canonical amino acids unveils masked epitopes in live neurons

Diogo Bessa-Neto[1*], Gerti Beliu[2,3*], Alexander Kuhlemann[2*], Valeria Pecoraro[1], Sören Doose[2], Natacha Retailleau[1], Nicolas Chevrier[1], David Perrais[1], Markus Sauer[2#], Daniel Choquet[1,4#]

[*] These authors contributed equally; [#] for correspondence: M.S. (m.sauer@uni-wuerzburg.de), D.C. (daniel.choquet@u-bordeaux.fr)

Containing:
Supplementary Figures 1-7

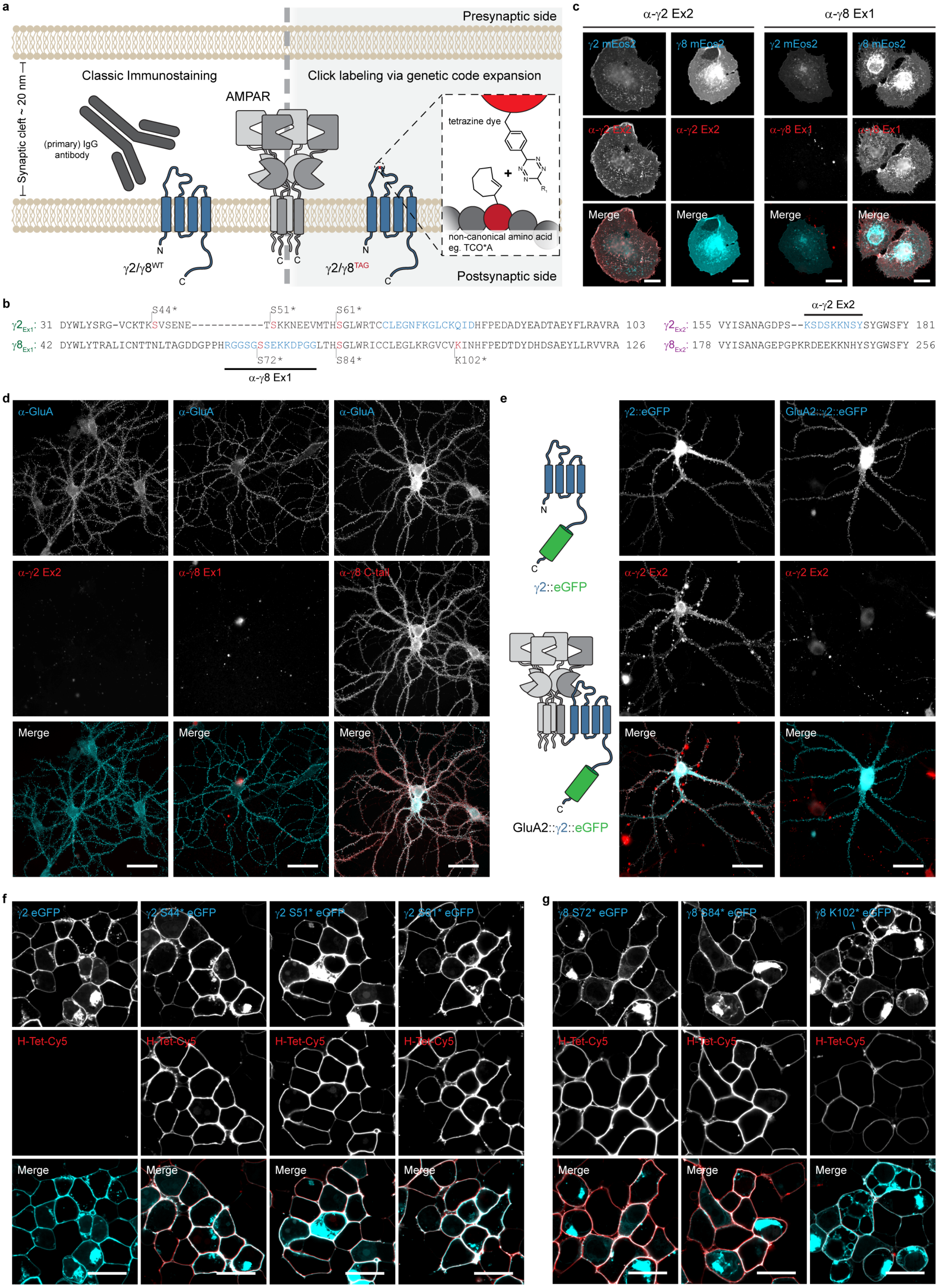

Supplementary Fig. 1: Unmasking the Ex1 loop of TARPs using bioorthogonal labeling. (a) Schematic illustration of the two approaches used to label the extracellular pool of γ2 and γ8. On the left side, the classic indirect immunostaining using whole IgG antibodies, and on the right, click chemistry

labeling via genetic code expansion. (**b**) Sequence alignment of the extracellular loops, Ex1 and Ex2, of γ2 and γ8 from *rattus norvegicus*. Amber substitution mutations are represented in red. The epitopes recognized by the antibodies are represented in blue. (**c**) Representative widefield images of fixed COS7 expressing either γ2 or γ8 bearing mEos2 live stained with the antibodies against the extracellular loops of γ2 (α-γ2 Ex2) or γ8 (α-γ8 Ex1). (**d**) Representative widefield images of fixed untransfected dissociated hippocampal neurons co-stained live with α-GluA1/2/3/4, and α-γ2 Ex2 (left), α-γ8 Ex1 (middle), or post-fixation/permeabilization with α-γ8 C-tail (right). (**e**) Left: schematic illustration of the eGFP-tagged γ2 constructs used, respectively γ2::eGFP (upper) and GluA2::γ2::eGFP (lower). Right: representative widefield images of fixed dissociated hippocampal neurons co-expressing Tet3G/tRNA^{Pyl}, and the doxycyline-inducible pTRE3G-BI γ2::eGFP (left) or pTRE3G-BI GluA2::γ2::eGFP, live stained with α-γ2 Ex2. (**f-g**) Representative confocal images of live HEK293T cells co-expressing PylRS/4xtRNA^{Pyl}, and (**f**) γ2::eGFP or ncAA-tagged γ2::eGFP or (**g**) ncAA-tagged γ8::eGFP in the presence of 250 μM TCO*A stained with 1.5 μM Pyr-Tet-ATTO643. Scale bar: (**c**, **f**, and **g**) 20 μm, (**d** and **e**) 50 μm. All representative images are representative of two or three independent preparations.

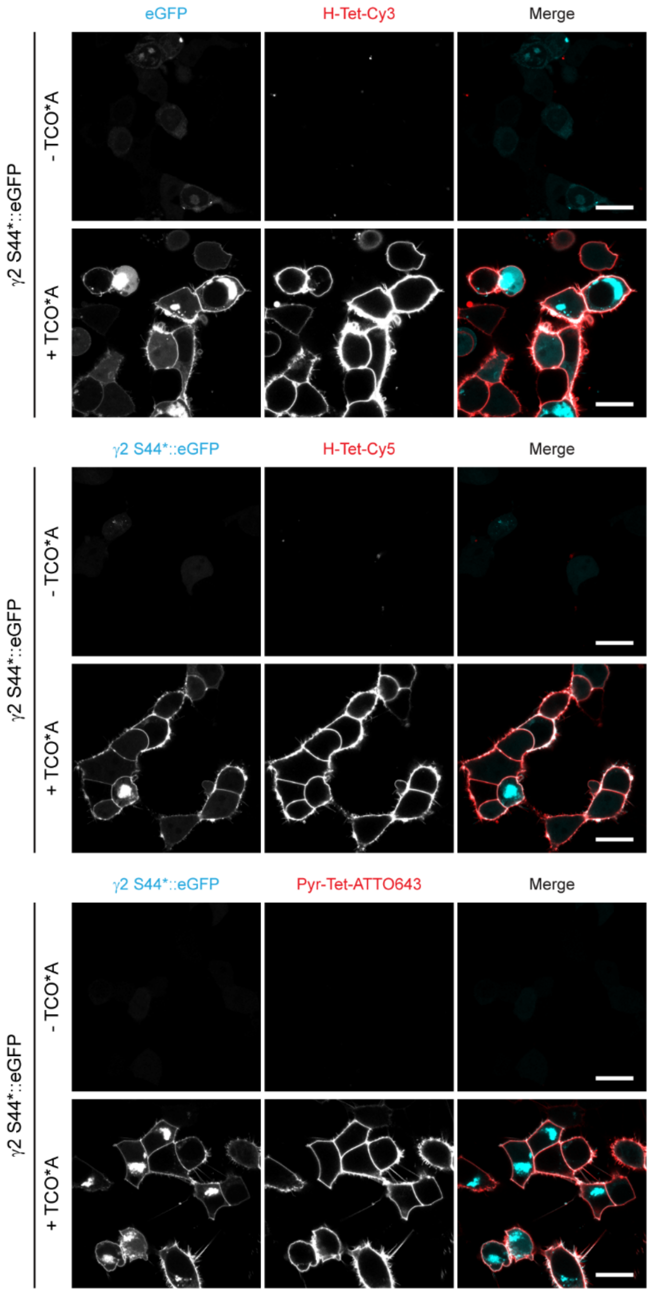

Supplementary Fig. 2: Bioorthogonal TARP click labeling with different tetrazine-dyes. Representative confocal images of living HEK293T cells co-expressing PylRS/4xtRNA^{Pyl} and γ2 S44*::eGFP without (-TCO*A) or with (+TCO*A) addition. Labeled with H-Tet-Cy3 (top), H-Tet-Cy5 (middle), and Pyr-Tet-ATTO643 (bottom). Scale bar: 20 μm. All representative images are representative of at least two independent preparations.

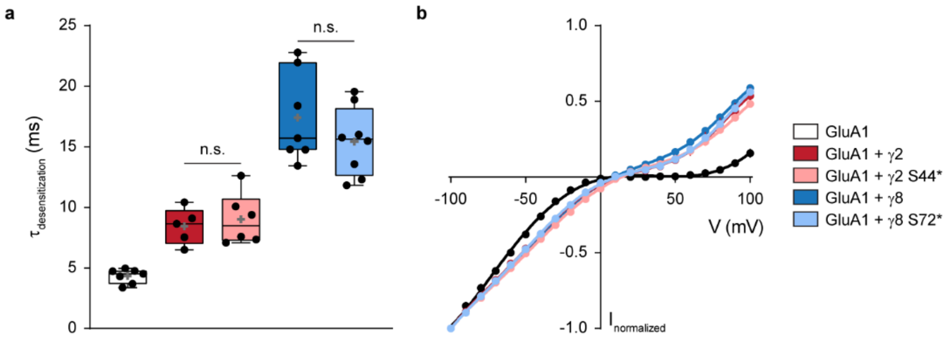

Supplementary Fig. 3: Incorporation of TCO*A within the EX1 does not affect TARP subtype-induced AMPAR modulation. (**a**) Desensitization rates in response to 100 ms of 10mM Glu applied to whole-cell patches from HEK293T cells co-expressing PylRS/tRNA^{Pyl}, GluA1 and, eGFP (τ_{des} = 4.34 ± 0.06 ms; 7 cells; black), γ2::eGFP (τ_{des} = 8.44 ± 1.50 ms; 5 cells; dark blue), γ2 S44*::eGFP (τ_{des} = 9.02 ± 2.14 ms; 6 cells; light blue), γ8::eGFP (τ_{des} = 17.41 ± 3.72 ms; 7 cells; dark red), and γ8 S72*::eGFP (τ_{des} = 15.43 ± 2.82 ms; 8 cells; light red). Box indicates 25th to 75th percentiles, whiskers represent max to min, with median represented as a centre line, and mean represented as a cross. Statistical difference was analyzed using one-way ANOVA with a Fisher's Least Significant Difference multiple comparisons test; n.s. specifies no significance. (**b**) I-V relationships for 10 mM Glu-evoked peak currents applied to whole-cell patches from HEK293T cells co-expressing PylRS/tRNA^{Pyl}, GluA1 and, eGFP (control; 7 cells; black), γ2::eGFP (5 cells; dark blue), γ2 S44*::eGFP (6 cells; light blue), γ8::eGFP (7 cells; dark red), and γ8 S72*::eGFP (8 cells; light red). Current are normalized to -100 mV. All data is pulled from three-to-four independent biological preparations. All data represent mean ± SD. Source data are provided as a Source Data file.

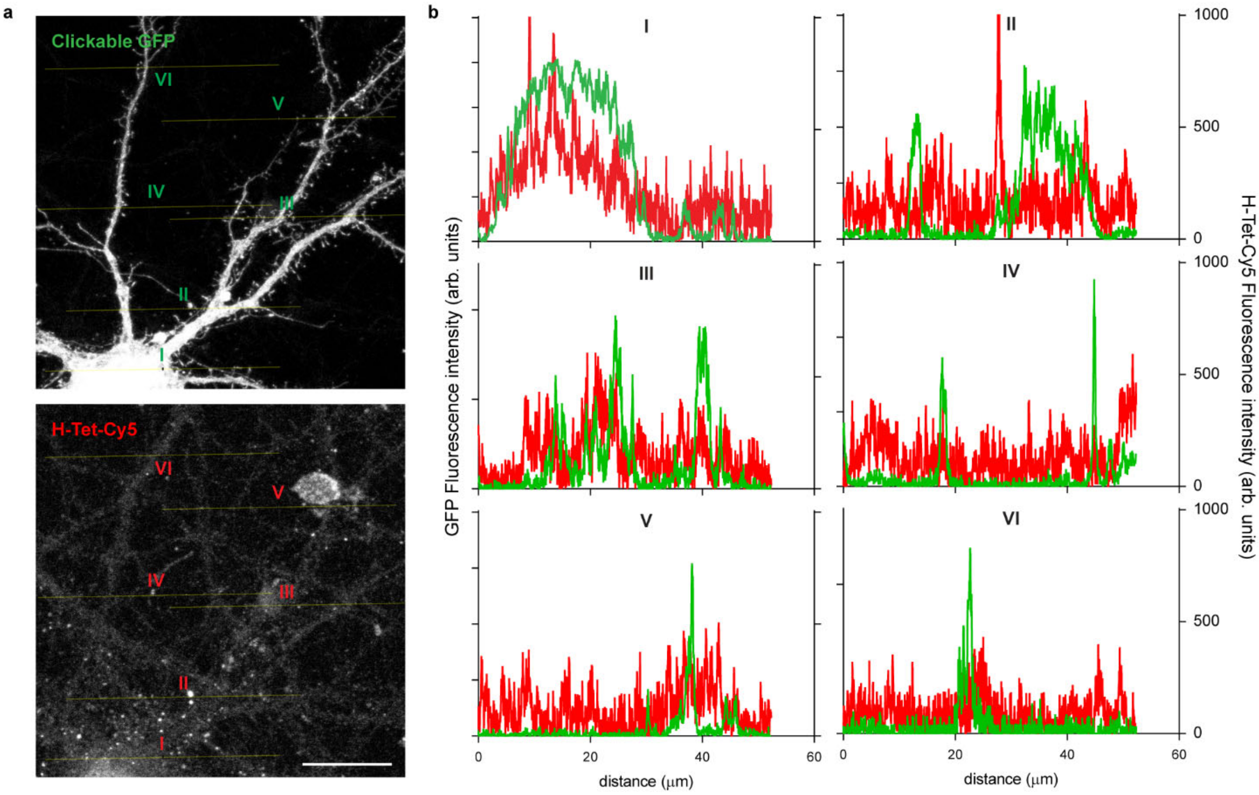

Supplementary Fig. 4: Absence of off-target surface labeling in PylRS/tRNA^{Pyl} transfected neurons. (**a**) Representative confocal image of fixed dissociated neurons co-transfected with intracellular clickable GFP, Tet3G/tRNAPyl and pTRE3G-BI PylRS at DIV15. Five days before H-Tet-Cy5 labeling, cells were treated with 250 μM TCO*A and 100 ng/mL doxycycline. 24hrs before labeling, an additional 125 μM TCO*A and 50 ng/mL was added to the cell media. Scale bar: 20 μm. (**b**) Line scan measurements of clickable-GFP (green) and H-Tet-Cy5 (red) across six different areas. On the left Y-axis (GFP fluorescence intensity) each tick interval corresponds to 1000 arbitrary units (arb. units), whereas on the right Y-axis (H-Tet-Cy5 fluorescence intensity) each tick interval corresponds to 500 arb. units. Expression of clickable-GFP indicates the success of the GCE experiment, while the transfected cell does not express more H-Tet-Cy5 labeling than non-transfected neighboring neurons, demonstrating the absence of any detectable off-target surface labeling in the absence of clickable surface protein. Representative images are representative of a single preparation.

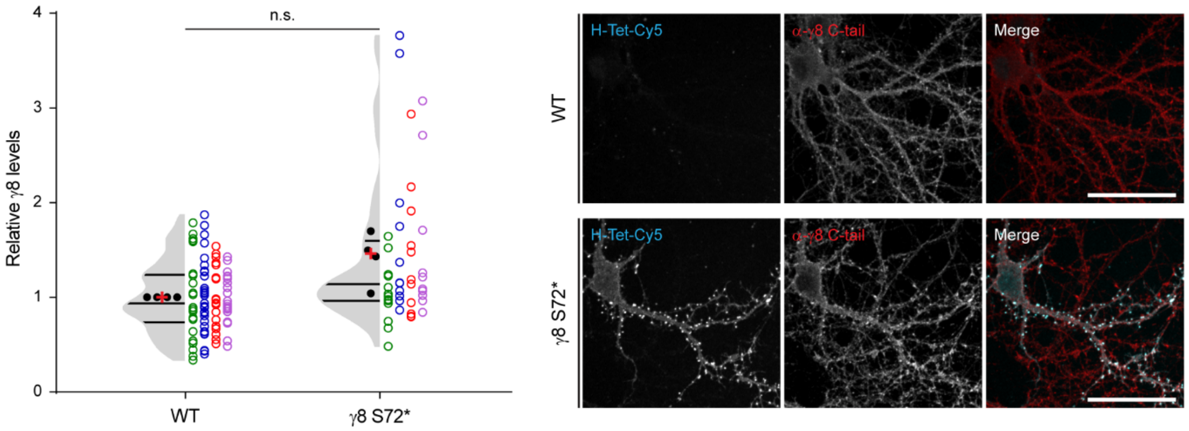

Supplementary Fig. 5: Comparison of γ8 levels between WT and γ8 S72*-positive neurons in dissociated hippocampal cultures. Normalized fluorescence intensity of α-γ8 C-tail antibody showed a slight increase of total γ8 protein in γ8 S72*-positive neurons (1.41 ± 0.27; $p < 0.0557$) in relation to non-transfected (WT) neurons. Circles on the right-half (green, blue, red and purple) of the violin plot represent the distribution of individual cells; 90 cells from four independent preparations. Violin indicates 25th to 75th percentiles (lower and upper lines, respectively), with median represented as a centre line, and mean represented as a cross; dots represent the mean value of relative γ8 levels per independent preparation. Statistical significance was analyzed using a two-tailed unpaired Welch's t test; n.s. specifies no significance. On the right side of the panel, representative spinning disk confocal images of WT (top) or γ8 S72*-positive (bottom) neurons labeled with α-γ8 C-tail antibody upon fixation and permeabilization of the cells. Scale bar: 50 μm. Source data are provided as a Source Data file.

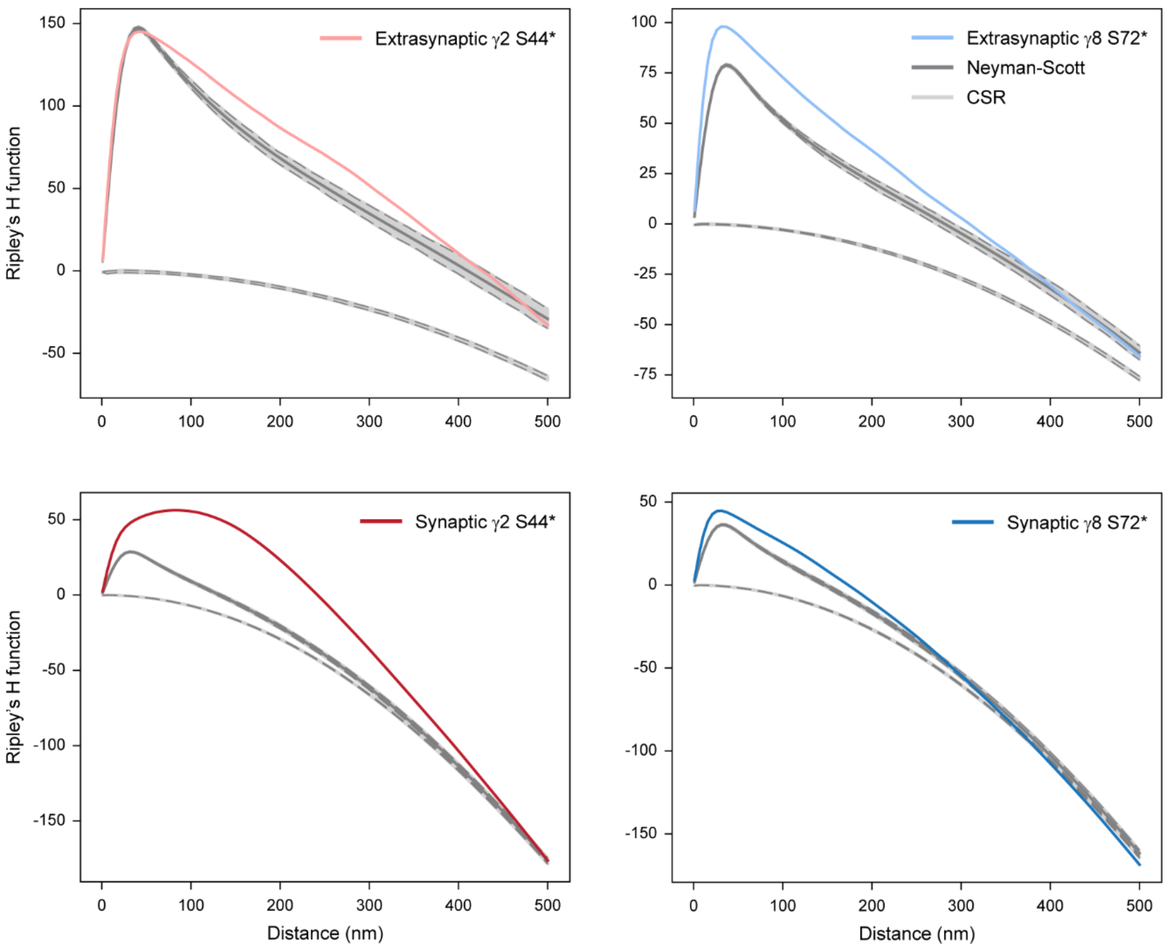

Supplementary Fig. 6: Ripley´s H function. γ2 S44* (red) as well as γ8 S72* (blue) show synaptic (dark) and extrasynaptic (light) random distributions of localization clusters with a size of ~20 nm. Only γ2 S44* (dark red) shows a non-random distribution in synaptic areas with a maximum at ~100 nm indicating cluster formation. Ripley´s H function from 100 replicates of simulated data with spatial distributions following complete spatial randomness (lower grey lines) or a clustered Neyman-Scott process (upper grey lines) in identical ROIs are displayed with 95% confidence intervals (dotted gray lines). Source data are provided as a Source Data file.

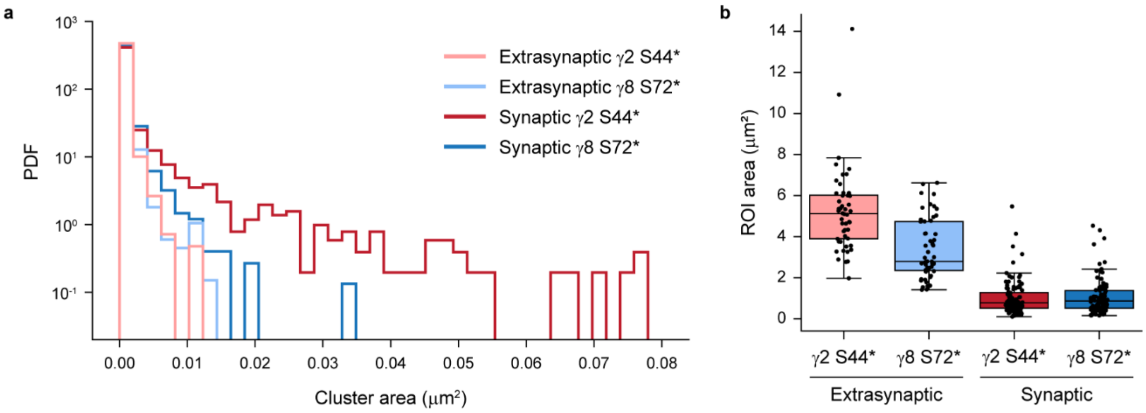

Supplementary Fig. 7: DBSCAN analysis of γ2 S44* and γ8 S72* localizations reveal the presence of clusters (>80nm) only for γ2 S44*in synapses. (**a**) Histograms of cluster areas displaying the probability density function (PDF) of the same cluster as analyzed in Fig. 5 (n = 2039, 3243, 2486, 3644 cluster from 50, 52, 104, 102 ROIs of five independent preparations for extrasynaptic γ2, extrasynaptic γ8, synaptic γ2, synaptic γ8, respectively). Extrasynaptic γ8 S72* (light blue), extrasynaptic γ2 S44* (light red) and synaptic γ8 S72* (dark blue) show similar cluster areas for all clusters with areas < 0.02 μm^2, whereas synaptic γ2 S44* (dark red) exhibit also clusters with cluster areas > 0.02 μm^2 corresponding to cluster diameter > ~80 nm. (**b**) Boxplots displaying selected ROI areas that were included in cluster analysis. ROI areas were expanded to be similar for synaptic γ8 S72* and γ2 S44* as well as for extrasynaptic γ8 S72* and γ2 S44* to ensure comparable cluster analysis between γ8 and γ2. Boxplots show lower to upper quartile and median values of the data with whiskers extending 1.5 * interquartile range. Source data are provided as a Source Data file.
