# Supplementary material for: Bioorthogonal labeling of transmembrane proteins with non-canonical amino acids unveils masked epitopes in live neurons
Source: Nat Commun. 2021 Nov 18;12:6715. doi: 10.1038/s41467-021-27025-w (PMC8602626; doi:10.1038/s41467-021-27025-w)
Supplement: Supplementary file 3 — Reporting Summary [file 41467_2021_27025_MOESM3_ESM.pdf]

nature research

Corresponding author(s): Markus Sauer
Daniel Choquet

Last updated by author(s): Oct 27 2021

Reporting Summary

Nature Research wishes to improve the reproducibility of the work that we publish. This form provides structure for consistency and transparency in reporting. For further information on Nature Research policies, see our Editorial Policies and the Editorial Policy Checklist.

Statistics

For all statistical analyses, confirm that the following items are present in the figure legend, table legend, main text, or Methods section.

n/a	Confirmed	
☐	☒	The exact sample size (*n*) for each experimental group/condition, given as a discrete number and unit of measurement
☐	☒	A statement on whether measurements were taken from distinct samples or whether the same sample was measured repeatedly
☐	☒	The statistical test(s) used AND whether they are one- or two-sided<br>*Only common tests should be described solely by name; describe more complex techniques in the Methods section.*
☐	☒	A description of all covariates tested
☐	☒	A description of any assumptions or corrections, such as tests of normality and adjustment for multiple comparisons
☐	☒	A full description of the statistical parameters including central tendency (e.g. means) or other basic estimates (e.g. regression coefficient) AND variation (e.g. standard deviation) or associated estimates of uncertainty (e.g. confidence intervals)
☐	☒	For null hypothesis testing, the test statistic (e.g. *F*, *t*, *r*) with confidence intervals, effect sizes, degrees of freedom and *P* value noted<br>*Give P values as exact values whenever suitable.*
☒	☐	For Bayesian analysis, information on the choice of priors and Markov chain Monte Carlo settings
☒	☐	For hierarchical and complex designs, identification of the appropriate level for tests and full reporting of outcomes
☐	☒	Estimates of effect sizes (e.g. Cohen's *d*, Pearson's *r*), indicating how they were calculated

Our web collection on statistics for biologists contains articles on many of the points above.

Software and code

Policy information about availability of computer code

Data collection	Electrophysiology data was acquired using the PatchMaster (v2x90.5) software. Widefield and spinning disk confocal images were acquired with MetaMorph (v7.8.10), confocal images (Fig. 3, Fig. 4, and Supplementary Fig. 4) were acquired with Leica Application Suite X (LAS X, v3.5.7) or (Fig. 1c,d, Supplementary Fig. 1f,g, and Supplementary Fig. 2) with ZENblack (v8.1.6.484), and FRET data was acquired with the LI-FLIM (v1.2.12) software.
Data analysis	Electrophysiology data was analysed using IGOR Pro (v6.0) software. Widefield and confocal images were analyzed using ImageJ (v1.53) software. FRET data was analyzed using LI-FLIM (v1.2.12) software. 2D Super-resolution images were reconstructed using the open source software rapidSTORM 3.36.<br>All the graphs and statistical tests were performed in the GraphPad Prism (v9.0).<br>Figures and illustrations were prepared using ImageJ (v1.53), and Adobe Illustrator (v23.1) software.<br>The code to perform cluster analysis will be made available for research and reproducibility purposes upon request by contacting the corresponding author. Requests will be answered within a week (m.sauer@uni-wuerzburg.de).

For manuscripts utilizing custom algorithms or software that are central to the research but not yet described in published literature, software must be made available to editors and reviewers. We strongly encourage code deposition in a community repository (e.g. GitHub). See the Nature Research guidelines for submitting code & software for further information.

Data

Policy information about availability of data

All manuscripts must include a data availability statement. This statement should provide the following information, where applicable:

- Accession codes, unique identifiers, or web links for publicly available datasets
- A list of figures that have associated raw data
- A description of any restrictions on data availability

The authors declare that data supporting the findings of this study are available within the main article and its supplementary information files. The raw microscopy data underlying the results will be made available upon request to the corresponding authors. Requests will be answered within a week. Source data are provided with this paper.

Field-specific reporting

Please select the one below that is the best fit for your research. If you are not sure, read the appropriate sections before making your selection.

☒ Life sciences ☐ Behavioural & social sciences ☐ Ecological, evolutionary & environmental sciences

For a reference copy of the document with all sections, see nature.com/documents/nr-reporting-summary-flat.pdf

Life sciences study design

All studies must disclose on these points even when the disclosure is negative.

Sample size	No sample size calculation was performed. Sample sizes for FRET experiments were estimated based on previous experience with data of this type (Charlotte Rimbault, et al. 2019. Nat Comm); experiments were performed in 2-5 biological independent preparations, per preparation at least 20 cells per condition were acquired. Sample sizes for electrophysiology experiments were based on previous experience (Veran, et al. 2012. Neuron); 5-8 cells from 2-3 biological independent preparations. Immunostaining were performed in 2-4 technical replicates from 2-3 independent biological experiments. For the spine-to-extraspine ratio analysis in dissociated neurons, 28 cells were acquired from 3 independent biological preparation (9-10 cells per preparation). Click-labeling of HEK cells, dissociated neurons, or slice cultures were performed in 2-4 independent biological experiments, with 2-4 technical replicates per biological preparation. For dSTORM experiments, data was pulled from 5 independent biological experiments. Presented samples sizes were considered sufficient based on the reproducibility of the results across multiple biological independent preparations.
Data exclusions	Only cells expressing sufficient levels of TARPs as detected by the camera were taken in consideration (average signal above 3000 counts measured on the tetrazine-dye intensity with a 16 bit camera) .<br>In the electrophysiology experiments, a cell from the condition PylRS/tRNAPyl + GluA1 + γ8::eGFP was not included for the KA/Glu analysis as we lost the cell when switching to the KA application.
Replication	All attempts of replication were successful from at least 2-5 independent biological preparations. The exact number is provided in the figure captions
Randomization	For in cellulo studies, samples (cells and slice cultures) were randomly allocated from one single pool into experimental groups corresponding to each treatment. Experiments could be reproduced at any timepoint within this study without affecting the results. This applies for all techniques including cLSM, FRET, dSTORM and electrophysiology experiments.
Blinding	Blinding was not performed as the same investigator was doing the group allocation (cell plating and treatment) and the data collection and analysis. Additionally, based on published data and pilot experiments, blinding of Gamma2 and Gamma8 was not relevant to the study because of the striking differential membrane expression of the two proteins obvious to the experimenter. To limit bias, all treatments and data collection were handled in parallel when physically possible, or, alternatively, randomly alternated.

Reporting for specific materials, systems and methods

We require information from authors about some types of materials, experimental systems and methods used in many studies. Here, indicate whether each material, system or method listed is relevant to your study. If you are not sure if a list item applies to your research, read the appropriate section before selecting a response.

Materials & experimental systems

n/a	Involved in the study	
☐	☒	Antibodies
☐	☒	Eukaryotic cell lines
☒	☐	Palaeontology and archaeology
☐	☒	Animals and other organisms
☒	☐	Human research participants
☒	☐	Clinical data
☒	☐	Dual use research of concern

Methods

n/a	Involved in the study	
☒	☐	ChIP-seq
☒	☐	Flow cytometry
☒	☐	MRI-based neuroimaging

Antibodies

Antibodies used

Mouse anti-GluA antibody was purchased from Synaptic Systems; cat. # 182 411C3.
Rabbit anti-Gamma8 antibody was purchased from Frontier Institute; cat. # TARPg8-Rb-Af1000.
Goat anti-mouse IgG AlexaFluor568 was purchased from ThermoFisher SCIENTIFIC; cat. # A-11004.
Goat anti-rabbit IgG AlexaFluor647 was purchased from ThermoFisher SCIENTIFIC; cat. # A-21244.
Rabbit anti-Gamma8 Ex1 was produced in-house.
Rabbit anti-Gamma2 Ex2 was produced in-house.

Validation

anti-GluA was raised against GluA2 but detects recombinant GluA1/2/3, specificity and suitability for ICC validated by supplier. No further information available; anti-Gamma8 (TARPg8-Rb-Af1000) specificity against mouse (others not tested) Gamma8 tested in WT and Gamma KO mouse, and suitable for WB/IHC validated by supplier. No further information provided. In-house produced Anti-Gamma8 Ex1 and anti-Gamma2 Ex2 specificity were validated using expression of recombinant Gamma2 and Gamm8 in COS-7 cells (Supplementary Fig. 1c). In the tested concentrations, each antibody showed specificity only towards the respective target protein. Suitable dilutions for all the antibodies were tested beforehand.

Eukaryotic cell lines

Policy information about cell lines

Cell line source(s)

HEK293T cells (ECACC, #12022001), COS-7 cells (ECACC, #87021302).

Authentication

Each cell line came directly from commercial sources that state that these cell lines are authentic. All information can be found in: HEK293T cells (https://www.sigmaaldrich.com/FR/fr/product/sigma/cb_12022001?context=product) and COS-7 cells (https://www.sigmaaldrich.com/FR/fr/product/sigma/cb_87021302?context=product).
We did not performed any in-house identification post arrival of aliquots.

Mycoplasma contamination

All cell lines were tested negative for mycoplasma contamination. Mycoplasma testing was conducted by a thrid-party institution (Eurofins) via qPCR-based assay from cell culture media.

Commonly misidentified lines (See ICLAC register)

No commonly misidentified cell lines were used in the study.

Animals and other organisms

Policy information about studies involving animals; ARRIVE guidelines recommended for reporting animal research

Laboratory animals

Gestant Sprague Dawley rat females at the age of 9 to 12 weeks old were purchased from Janvier Labs, Saint-Berthevin, France. Wild-type C57Bl6/J mice of both sexes at P5-7. Animals were housed at PIV-EOPS facility of the IINS under a 12 hour light/dark cycle at normal room temperature (22ºC) and humidity between 40-70% (typically 60%) with unrestricted access to food and water.

Wild animals

Study did not involve wild animals.

Field-collected samples

Study did not involve samples collected from the field.

Ethics oversight

All experiments were performed in accordance with the European guidelines for the care and use of laboratory animals, and the guidelines issued by the University of Bordeaux animal experimental committee (CE50; Animal facilities authorizations A3306940 and A33063941).

Note that full information on the approval of the study protocol must also be provided in the manuscript.
